# Magnetic Resonance Imaging in Animal Models of Alzheimer’s Disease Amyloidosis

**DOI:** 10.3390/ijms222312768

**Published:** 2021-11-25

**Authors:** Ruiqing Ni

**Affiliations:** 1 Institute for Biomedical Engineering, ETH Zurich & University of Zurich, 8093 Zurich, Switzerland; ni@biomed.ee.ethz.ch; 2 Institute for Regenerative Medicine, University of Zurich, 8952 Zurich, Switzerland

**Keywords:** Alzheimer’s disease, amyloid-β, animal model, diffusion tensor imaging, functional imaging, magnetic resonance imaging, magnetic resonance spectroscopy

## Abstract

Amyloid-beta (Aβ) plays an important role in the pathogenesis of Alzheimer’s disease. Aberrant Aβ accumulation induces neuroinflammation, cerebrovascular alterations, and synaptic deficits, leading to cognitive impairment. Animal models recapitulating the Aβ pathology, such as transgenic, knock-in mouse and rat models, have facilitated the understanding of disease mechanisms and the development of therapeutics targeting Aβ. There is a rapid advance in high-field MRI in small animals. Versatile high-field magnetic resonance imaging (MRI) sequences, such as diffusion tensor imaging, arterial spin labeling, resting-state functional MRI, anatomical MRI, and MR spectroscopy, as well as contrast agents, have been developed for preclinical imaging in animal models. These tools have enabled high-resolution in vivo structural, functional, and molecular readouts with a whole-brain field of view. MRI has been used to visualize non-invasively the Aβ deposits, synaptic deficits, regional brain atrophy, impairment in white matter integrity, functional connectivity, and cerebrovascular and glymphatic system in animal models of Alzheimer’s disease amyloidosis. Many of the readouts are translational toward clinical MRI applications in patients with Alzheimer’s disease. In this review, we summarize the recent advances in MRI for visualizing the pathophysiology in amyloidosis animal models. We discuss the outstanding challenges in brain imaging using MRI in small animals and propose future outlook in visualizing Aβ-related alterations in the brains of animal models.

## 1. Introduction

The two core pathological hallmarks of Alzheimer’s disease (AD) are extracellular amyloid-beta (Aβ) plaques and intracellular neurofibrillary tangles, resulting from the abnormal accumulation of misfolded Aβ and tau [1]. Aβ plays a central role in the pathogenesis of AD and downstream pathophysiological events [2]. The pathophysiological changes in AD start many years before the onset of clinical symptoms [3]. Recent advances in diagnostic imaging have provided insights into the time course of AD pathology, including Aβ, tau, and neuroinflammation, in patients and in animal disease models [4,5]. Magnetic resonance imaging (MRI) is widely used both in clinical settings for assisting in the diagnosis toward precision medicine and in preclinical research in small-animal models. Structural MRI for assessing the neurodegeneration (brain atrophy) in the ATN framework has offered a valuable tool for early and differential diagnosis of AD and for disease staging [4,6]. Moreover, multiplex MRI sequences, such as diffusion tensor imaging (DTI) for white matter integrity assessment, resting-state (rs) functional MRI for functional connectivity analysis [7,8], as well as arterial spin labeling (ASL) for cerebral perfusion measurement, have emerged as potential diagnostic biomarkers for AD.

Several generations of animal models of AD amyloidosis have been developed. The animal models, including transgenic APPswe, APP/PS1, APP23, and J20 mouse models; McGill-R-Thy1-APP rat models [9,10,11,12,13]; and 2^nd^ generation App^NL-G-F^ and App^hu/hu^ knock-in and 3^rd^-generation mouse models, overexpress human *Amyloid Precursor Protein (APP)* and/or *Presenilin (PS)* [14,15,16,17]. The Aβ deposits, both parenchymal plaques and cerebral amyloid angiopathy (CAA), first accumulate in the neocortex, limbic regions and later in the subcortical brain regions. The speed of pathology development in these animal models depends on *APP* expression levels and mutations. Aβ pathology, especially the most neurotoxic oligomeric Aβ, plays a crucial role in the disease pathogenesis in animal models and leads to downstream gliosis, neuronal loss, and functional and cognitive impairment [18,19]. In addition, models harboring both Aβ and tau pathology, such as 5 × FAD and 3 × Tg mice and TgF344 rats, have been commonly used [20,21,22]. In this review, we summarize recent advances in MRI, contrast agents, and MR spectroscopy in probing the alterations in brains of AD amyloidosis animal models. We outline the outstanding challenges and provide an outlook for further development of preclinical MR in animal models of AD amyloidosis.

## 2. Aβ Imaging

**Figure 1:**
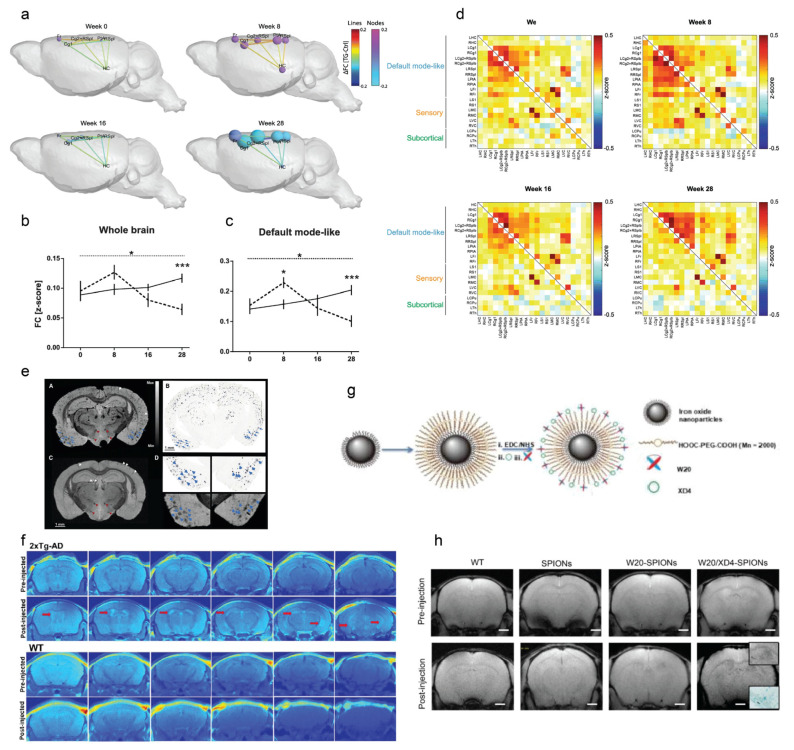
Functional MRI and amyloid imaging in amyloidosis animal models. (**a**–**d**) Aberrant functional connectivity (FC) in the default mode-like network (DMN) in the Tet-Off APP mice with doxycycline treatment. (**a**) difference in FC within (nodes) and between (lines) regions in the DMN over time: weeks 0, 8, 16, and 24. The inter-node FC difference is represented by the lines, with the color scale illustrating the actual FC difference between Tet-Off APP and Ctrl, with orange indicating a stronger connection in the TG mice. The intra-node size represents the difference in the average FC of a specific region from all other regions inside DMN. (**c**) ROI-based FC analysis. FC matrices show the average z-transformed functional connectivity (zFC) for Ctrl (supra-diagonal) and TG (sub-diagonal) animals at weeks 0, 8, 16, and 28 post doxycycline treatment. Each square indicates the zFC between a pair of ROIs. The color scale represents the connectivity strength, with white indicating a low zFC and red/blue indicating positive/negative zFC values. (**d**) Average FC within each network, the mean FC (z-score) over time for both groups in the whole brain, and the default-mode-like network; the dashed line corresponds to the TG group and the full line to the Ctrl group. * *p* < 0.05; *** *p* < 0.001. Reproduced from [75] with permission from Springer Nature. (**e**) MRI amyloid imaging. (A) T_2_*-weighted image at 16.4 T of a 30-month-old transgenic APP23 mouse and (B) corresponding amyloid histology; (C) T_2_*-weighted image at 16.4 T of the control mouse. Mammillothalamic tract and perifornical nucleus (red arrowheads). (D) Higher magnification of A and B of single amyloid plaques (blue arrowheads). Reproduced from [24] with permission from Society of Nuclear Medicine and Molecular Imaging. (**f**) In vivo T_1_-weighted MR-pseudocolor-mapped images of 6-month-old double Tg-AD and age-matched wild-type mice before and after i.v. injection of the cyanine–Gd(III) complex at different depths in which the images were taken 10 μm apart 90 min post-injection of the probe on a 7.0 T MR scanner. Reproduced from [37] with permission from American Chemical Society. (**g**,**h**) W20/XD4-SPIONs characterization (**g**). The carboxyl of PEG on the paramagnetic iron oxide nanoparticles (SPIONs) was activated with EDC and NHS. SR-A activator XD4 and oligomer-specific scFv antibody W20 were conjugated to the nanoparticles. (**h**) In vivo T_2_*-weighted images of the probe distribution in AD mouse brains after intravenous injection of W20/XD4-SPIONs, W20-SPIONs, and SPIONs. Boxed regions are shown at a higher magnification or stained by Prussian blue. Scale bar, 1 mm. Reproduced from [56] with permission from Dovepress.

In vivo Aβ detection and longitudinal monitoring in mouse models of AD amyloidosis have provided insights into the disease mechanisms and treatment effects. MRI detection of Aβ deposits has also been developed with or without using contrast agents (Table 1). Aβ imaging using MRI without contrast agents has been developed by exploring the changes in tissue proton MR properties, such as T_2_, T_2_* [23,24] (Figure 1e), magnetic susceptibility, magnetization transfer imaging [25,26], and chemical exchange-sensitive spin-lock (CESL) imaging [27]. The T_2_ relaxation time was found to be associated with Aβ pathology in several amyloidosis mouse models [28,29,30]. As iron, copper, and zinc accumulate inside the Aβ plaques [31], susceptibility-weighted imaging (SWI) and quantitative susceptibility mapping (QSM) have been used to detect Aβ aggregates and iron accumulation in brains of APP/PS1 and Tg-SwDI mice [32,33]. A few exogenous MRI contrast agents that can specifically bind to Aβ have been developed, including the following:

(1) Gadolinium (Gd) based: Gd-diethylenetriamine pentaacetate (DTPA)-Aβ1-40, Gd-DTPA-K6Aβ1-30, cyanine-conjugated Gd (III) complex, Gd-pF(ab’)24, and liposomal macrocyclic Gd-ADx-001 [34,35,36,37,38,39] (Figure 1f).

(2) Superparamagnetic iron oxide (SPIO) based: APP-SiMag, ultrasmall SPIO-polyethylene glycol-Aβ1-42.B, and IgG4.1 NP bifunctional ultrasmall SPIO [40,41,42,43,44]. Dudeffant et al. demonstrated the detection of compact Aβ plaques as well as CAA and microhemorrhages in five mouse lines (APP_SL_/PS1_M146L_, APP/PS1_dE9_, APP23, APP_SwDI_, and 3 × Tg) and in AD human brains using DOTAREM^®^ (Gd-DOTA) at 7 T MRI even around a diameter of 25 μm [45].

(3) Manganese (Mn) based: Mn-oxide-nanoparticle-conjugated HMON-Aβ40 [28], Mn chloride [46], monocrystalline iron oxide nanoparticles [35], and sialic-acid-coated bovine serum albumin magnetic nanoparticle [47], have been reported for Aβ deposit detection.

(4) ^19^F and ^1^H MRI using contrast agents such as small chemical dyes FSB, TFMB, bovine serum albumin@FDQDs, Shiga-Y5, and Shiga-Y51 have also been reported for in vivo Aβ imaging in animal models [37,48,49,50,51,52].

Moreover, several contrast agents specific to Aβ oligomer (antibody-based or chemical probe) have been reported. Viola et al. reported using 12–16 nm Fe_3_O_4_ magnetic nanostructures (MNS) conjugated with Aβ-oligomer-specific antibody NU4 for detecting Aβ oligomers in mouse brains [53]. Rozema et al. reported Aβ-oligomer-specific antibody-based ACU193-MNS for detecting the Aβ oligomer levels in rabbits by using MRI [54]. As the size of the antibody hinders the permeability of its blood–brain barrier (BBB), one strategy to facilitate the antibody delivery is to link a fraction of the antibody with a transferrin [55] or scavenger receptor. Liu et al. demonstrated in vivo detection in APP/PS1 mice by using W20/XD4-SPIO nanoparticles conjugated with an Aβ-oligomer-specific single-chain variable fragment (scFv) and a scavenger receptor [56] (Figure 1g–h). Chen et al. and Dong et al. reported curcumin-derivative-conjugated magnetic nanoparticles (Cur-MNPs) for in vivo imaging of Aβ with high contrast in APPswe and 5 × FAD mice [57,58].

## 3. Functional Imaging

Synaptic impairment, aberrant excitatory neuronal activity, gamma oscillations, and disrupted circuit are early features in amyloidosis animal models [68,69,70,71]. Clusters of hyperactive neurons are observed in the vicinity of Aβ plaques in APP mouse models [72]. There is a vicious cycle of Aβ-dependent neuronal hyperactivation initiated by the suppression of glutamate reuptake [73]. Neurovascular uncoupling and impaired cerebral blood flow (CBF) have been demonstrated by MRI and optical imaging modalities [74].

### 3.1. Manganese-Enhanced (ME) MRI

Both the neuronal tracing MEMRI and the activity-induced MEMRI methods for detecting active neural regions during a task or a stimulation, independent of hemodynamics, have been developed [76,77]. MEMRI is based on the following properties of manganese ions (Mn^2+^): Mn^2+^ is a paramagnetic ion that shortens the T_1_ relaxation time, and is an excellent T_1_ contrast agent; as a calcium ion (Ca^2+^) analog, Mn^2+^ can enter via voltage-gated Ca^2+^ channels inside neurons; and Mn^2+^ can cross synapses to neighboring neurons and along axon via microtubule-dependent axonal transport [76,77]. Activity-induced MEMRI has been applied in APPswe, APP × PS1-Ki, CVN-AD, J20, and 5 × FAD mice and TgF344 rats [46,78,79,80,81] (Table 2). Most MEMRI studies found hyperactivation and functional abnormalities in the APP animal models. However, studies have also reported that activity-induced MEMRI cannot detect hyperactivation in the APP × PS1-Ki mouse [78]. An MEMRI study in TetO/APP_SwInd_ with overexpression of *APP* specifically in olfactory neurons was shown to detect laminar changes and neurodegeneration in the olfactory bulb [82]. Neuronal tracing studies with MEMRI by direct injection of manganese chloride solution into the mouse brain region enable the detection of impaired axon transport. Intranasal administration of Mn showed decreased axonal transport rates in the olfactory system prior to Aβ plaque formation in a mouse model in APPswe and 3 × Tg mice [83,84,85] and in the hippocampus-basal forebrain pathway in TetO/APP_SwInd_ mice [86].

### 3.2. Resting-State Functional MRI

fMRI has enabled a better understanding of brain activity and has become a workhorse in neuroimaging [7] and a potential early biomarker for neurodegenerative diseases. Blood-oxygen-level-dependent (BOLD) signals from rs-fMRI have been widely used as a readout for brain function [87]. Early hypersynchrony of BOLD resting-state networks in the telencephalic, interhemispheric, and hippocampal regions, as well as the fornix, has been reported in amyloidosis mouse models, providing a predictive value for later cognitive dysfunction [81,88,89,90,91,92,93,94,95] (Table 2). Latif-Hernandez et al. reported that subtle behavioral changes and increased prefrontal-hippocampal network synchronicity in APP^NL−G−F^ mice occur prior to the Aβ plaque deposition [96]. Ben-Nejma et al. reported that an increased level of soluble Aβ causes early aberrant brain network hyper-synchronization in the default mode network (DMN)-like brain regions in inducible transgenic Tet-Off APP animal model at 8 weeks post doxycycline treatment; hypo-synchronization was detected by rs-fMRI at 20 weeks post doxycycline treatment in mature-onset Tet-Off APP mice [75] (Figure 1a–d). Another study reported diminished functional connectivity in APP/PS1 mice compared to wild-type littermates [97]. Canter et al. demonstrated that the DMN is affected early, at 4 months-of-age prior to the limbic system, along with a network-specific amyloid progression in 5 × FAD mice harboring both Aβ and tau pathologies [98]. Tudela et al. reported an early alteration in the anterior DMN subnetwork in TgF344 rats compared to wild-type rats by rs-fMRI using independent component analysis [99].

### 3.3. Arterial Spin Labelling (ASL)

**Figure 2:**
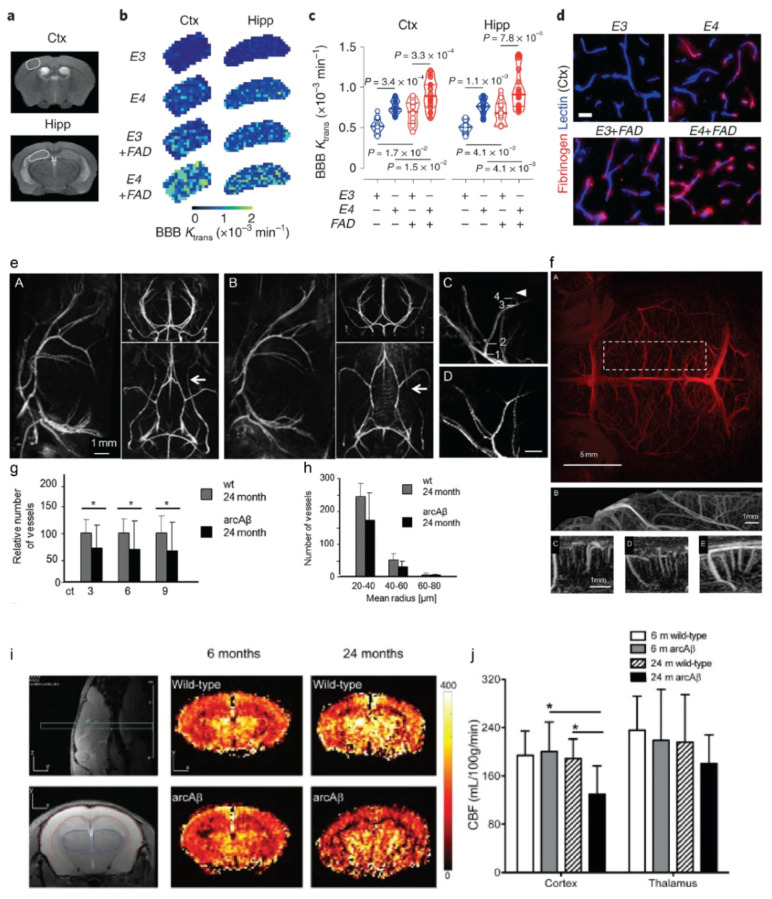
MRI of blood–brain-barrier permeability and cerebrovasculature in amyloidosis animal models. (**a**–**d**) T_2_-weighted scans displaying regions of interest: primary somatosensory cortex (Ctx) and hippocampus (Hipp). Representative K^trans^ maps (**b**) and values (**c**) in the Ctx and Hipp in APOE3 (E3, hollow blue circles), APOE4 (E4, solid blue circles), APOE3; 5 × FAD (E3 + FAD, hollow red circles), and APOE4; 5 × FAD (E4 + FAD, solid red circles) mice generated from dynamic contrast-enhanced MRI scans. (**d**) Fibrinogen+ perivascular capillary deposits (red) in the Ctx. Blue, lectin+ endothelial profiles; scale bar, 20 μm. Reproduced with permission from [141] from Springer Nature. (**e**–**h**) High-resolution magnetic resonance angiography (MRA). (**e**) Time-of-flight-MRA intra- and extracranial vasculature of 24-month-old wild-type and arcAβ mice (A, B) in sagittal, axial, and horizontal views. Flow voids are seen in extracranial vessels (white arrows). Sections of maximum intensity projections (MIPs) of the anterior cerebral artery of a 4- and a 24-month-old (C,D) wild-type control mouse. Scale bar, 1 mm. (**f**) Contrast-enhanced MRA; MIPs derived from a 3D stack of difference images viewed in horizontal (A), sagittal (B–D), and axial (E) orientations. (**g**,**h**) Semiautomated analysis of intracortical vessel density. (**g**) Significant decrease in the number of vessels was observed in a 24-month-old arcAβ mouse compared with a wild-type mouse corresponding to 3, 6, and 9 pixels, (* *p* < 0.05, repeated-measures ANOVA, and Tukey‘s test). (**h**) Number of vessels categorized according to their estimated vessel radius when the connectivity threshold was set to 3. Reproduced with permission from [156] from Society of Neuroscience. (**i**,**j**) Regional hypoperfusion in aged arcAβ mice assessed by arterial spin labeling MRI. Anatomical position of perfusion MRI and T_2_-weighted scan in the sagittal view. Representative coronal cerebral blood flow (CBF) map of 6- and 24-month-old wild-type littermate; (**j**) reduced CBF in the cortex of a 24-month-old arcAβ mouse compared to an age-matched wild-type mouse and a 6-month-old arcAβ mouse; * *p* < 0.05, one-way ANOVA with post hoc correction. Reproduced from [105] with permission from Elsevier.

ASL is used to quantify tissue blood flow or perfusion and is also routinely performed in the clinical setting [100]. Cortical hypoperfusion by using ASL has been reported in APP/PS1, Tg-SwDI, arcAβ, APPswe, and APP23 mice [24,101,102,103,104,105,106,107,108,109], as well as in 3 × Tg, bigenic, and 5 × FAD mice harboring both Aβ and tauopathy (Table 2; Figure 1f). Reduced cortical CBF was observed in the aged arcAβ mice (24 months-of-age) compared to aged wild-type mice and young arcAβ mice (Figure 2i,j). Cruz Hernández et al. demonstrated that neutrophil adhesion in brain capillaries impairs the CBF and that treatment using anti-neutrophil marker antibody reverses the CBF reduction and memory impairment in APP/PS1 mice [101].

### 3.4. Cerebrovascular Reactivity Measurement

Vasodilatory-stimulus-challenged fMRI assesses the cerebrovascular reactivity based on the cerebral hemodynamic changes and reflects the vascular reserve and autoregulatory function [157]. Different vasodilatory stimuli, including carbon dioxide, breath-hold task (in humans), and acetazolamide, have been used in animal models [133,137]. In addition, impaired cerebrovascular reactivity assessed by using fMRI with carbon dioxide as the stimulus has been reported in patients with mild cognitive impairment and AD [157,158,159]. In an amyloidosis mouse model, Gd- or SPIO-based contrast agents (e.g., Endorem) were intravenously injected through a tail vein to monitor the signal alterations due to the administration of acetazolamide [137]. Reduced cerebrovascular reactivity has been reported in APP/PS1, arcAβ, APPswe, APP23, J20, PDAPP, and BiAT mice compared to the wild-type mice [43,104,111,117,136] (Table 2)**.**

## 4. Neurochemical Changes Detection

### 4.1. Magnetic Resonance Spectroscopy (MRS)

MRS has been shown to detect the distinct metabolic profiles in APP/PS1 [121,147,148,149,150,151], and APPswe mice [115,154] compared to wild-type mice. Several studies have reported a reduced N-acetylaspartate/creatine ratio [148,149] and a lower glutamate level in APP/PS1 mice compared to wild-type mice [121]. Different metabolic profiles have also been demonstrated in animal models harboring both Aβ and tau pathologies, including 3 × Tg mice [153], 5 × FAD mice [152], APP/PS2/Tau mice [155], and TgF344 rats [146] (Table 2). Lee et al. demonstrated a 35% decrease in the availability of metabotropic glutamate receptor 5 measured by PET; a decrease in the levels of glutamate, N-acetylaspartate, and taurine; and an increase in the level of lactate by ^1^H MRS in 5 × FAD mice compared to wild-type at 5 months-of-age [152]. Using longitudinal ^1^H MRS, Chiquita et al. showed an early loss of taurine in the hippocampus in 3 × Tg mice compared to wild-type mice [153]. Micotti et al. reported striatal atrophy and increases in the level of myo-inositol in TASTPM and APP/PS2/Tau mice compared to wild-type mice, respectively [155].

### 4.2. Chemical Exchange Saturation Transfer (CEST)

Molecular MR imaging based on CEST offers improved sensitivity and can detect changes in the levels of glucose, glutamate, creatine, and myoinositol. Endogenous CEST measurements have been reported in amyloidosis models: Glucose CEST MRI detects unlabeled endogenous glucose at physiologically relevant concentrations using proton-only MRI scanners (Table 2). Using glucose CEST MRI, Tolomeo et al. demonstrated a reduced cerebral 2-deoxy-D-glucose uptake in APP23 mice compared to wild-type mice [143]. Igarashi et al. demonstrated a reduced level of glutamate measured by using glutamate CEST, as an indicator of synaptic dysfunction, in the parietal cortex but not in the hippocampus of 5 × FAD mice compared to wild-type mice [129]. Using creatine CEST MRI, Chen et al. demonstrated a reduced level of creatine in the cortex and corpus callosum of APP/PS1 mice compared to wild-type mice at 6 months-of-age [145]. Chen et al. showed a reduced saturation transfer difference for the composite protein amide proton in APP/PS1 mice compared to the age-matched wild-type mice [144].

## 5. Cerebrovascular Imaging

Accumulating evidence indicates the vascular contribution to cognitive impairment and in the development of AD [160,161]. Impaired cerebral vasculature has also been reported in various amyloidosis amyloid models with parenchymal Aβ plaques and different levels of CAA [162,163].

### 5.1. Susceptibility Weighted Imaging (SWI)

The presence of iron can be detected by MRI due to its effect on the surrounding tissue, giving rise to detectable changes in transverse T_2_ relaxation by using T_2_* and in susceptibility by using SWI and QSM (Table 3). A previous X-ray microscopy study reported the presence of particulate and crystalline iron inside the dense Aβ plaque core in the APP/PS1 mouse brain [164]. Beckmann et al. showed microhemorrhages in β-secretase inhibitor-treated APP23 mice by using T_2_*-weighted imaging [165]. A recent study by Maniskas et al. demonstrated a gender difference in the number of cerebral microbleeds by using a T_2_* sequence in Tg-SwDI mice (with a higher load of microbleeds in female mice) [166]. SWI and QSM have been performed in arcAβ, APP/PS1, and CVN-AD mice at 9.4 T [81,112,167,168,169]. McIntosh et al. showed that iron accumulation detected by SWI contributes to the altered cerebral metabolism and cognitive impairment in APP/PS1 mice [168].

### 5.2. MR Angiography (MRA)

MRA has been widely used in clinical settings as well as in small-animal imaging for assessing the cerebrovasculature abnormalities. Intracranial stenosis assessed by using MRA was observed in patients with cognitive impairment and AD [209,210]. Both time-of-flight and contrast-enhanced (CE)-MRA have been applied in amyloidosis animal models (Table 3). The detection of vascular alterations by in vivo MRA and histology has been reported in APP/PS1, arcAβ, APP/PS1, and APP23 mice [30,115,134,136,156]. Klohs et al. demonstrated a reduced density and remodeling of cerebral microvasculature in aged arcAβ mice compared to wild-type mice by using CE-MRA [156] (Figure 2e–h). MR Q mapping assisted with SPIO further showed a reduced level of microvessel density in the brains of arcAβ mice compared to wild-type mice, correlating with the levels of Aβ pathology [200]. Several MR techniques have been developed recently for assessing the cerebrovasculature and blood-brain barrier integrity [141, 201, 202]. Chang et al. reported using diffusion-weighted imaging assisted with monocrystalline iron oxide nanoparticle for assessing the abnormalities in the vessel size index, diameter, density, mean vessel-weighted image, and blood volume fraction in 5 × FAD mice compared to wild-type mice [203]. Leaston et al. showed early vascular abnormalities in APOE4 knock-in rats compared to wild-type rats by using quantitative ultra-short time-to-echo (QUTE) CE-MRI [202]. MR elastography has been used to detect the impaired cerebral viscoelastic properties in 5 × FAD, APP/PS1, and APP23 mice [206,207,208]. Montagne et al. demonstrated brain cerebrovascular inflammation by using T_2_*-weighted MRI assisted with micro-sized particles of iron oxide (MPIO) targeting vascular cell adhesion molecule 1 (VCAM-1) in APP/PS1 mice [201].

## 6. Structural Imaging

### 6.1. Volumetric Imaging for Brain Atrophy

In vivo MRI using T_1_ and T_2_ scans and histological evaluation has identified differences in the entire brain or regional brain volumes between amyloidosis animal models and wild-type littermates, including APP T714I, APP/PS1, APP/PS1 KI, 3 × Tg, and TASTPM mice and McGill-R-Thy1-APP rats [117,154,171,172,173,174,179,180,211] (Table 3). Delatour et al. reported global atrophy and an enlarged cerebrospinal fluid (CSF) space in the posterior brain areas and the midbrain areas in fiber tracts in APP/PS1 mice compared to wild-type mice [172]. Badhwar et al. demonstrated an impaired spatial learning/memory-induced volume increase in the hippocampus of APP/J20 mice compared to wild-type mice [170].

### 6.2. DTI

Extensive myelin loss was observed in amyloidosis animal models as well as in individuals with AD by in vivo imaging as well as by using histopathological investigations [194,212,213]. Recent studies have shown that myelin loss drove Aβ deposition and that enhancing myelin renewal in turn alleviated the cognitive deficits in APP/PS1 [214] and in 5 × FAD mice [215]. Impairment in white matter integrity has been detected by using in vivo and ex vivo DTI MRI in APPswe, APP/PS1, TgCRND8, APP^NL-G-F^, 3 × Tg, CVN-AD, and 5 × FAD mice compared to wild-type mice [81,91,95,184,185,186,189,191,196,216,217,218] (Table 3). In these studies, the DTI abnormalities were detectable prior to the anatomical changes becoming visible in structural MRI. Reduced fractional anisotropy (FA) and both reduced/increased radial diffusivity (RD) were reported in aged APPswe mice compared to wild-type mice [186,188,193]. In addition to DTI, diffusion kurtosis imaging (DKI) and quantitative magnetization transfer imaging (qMTI) have detected hippocampal alterations in APP/PS1 and APPswe mice compared to wild-type mice [182,184]. Falangola et al. and Zhou et al. reported basal forebrain cholinergic abnormalities, detected by DTI and DKI, in 3 × Tg mice compared to wild-type mice [183,192]. Kastyak-Ibrahim et al. reported a lack of white matter pathology in the same mouse line [198]. Reduced FA has also been reported in the gray matter in the brain of 3 × Tg, APP/PS1, and APP23 mice [91,121,188,194,195,196,219]. Colon-Perez et al. reported reduced levels of FA and RD and increased orientation dispersion and intracellular volume fraction in the white matter and hippocampus of TgCRND8 mice compared to wild-type mice, by using in vivo DTI MRI at 11.1 T and the neurite orientation dispersion and density imaging (NODDI) analysis pipeline [113]. In the CVN-AD mice, the white matter impairment was also associated with microglia activation [95].

## 7. Discussion

With the increased availability of and technological development in small-animal MRI, there is a rapid advance in molecular, functional, and structural imaging in AD amyloidosis animal models. Preclinical brain imaging is facing the unique challenge of the gap between man and mouse/rat models. Species differences in size, cell type, structure, circuit, the levels of protein expression, and metabolism has hindered the translation of imaging biomarkers from small animals to humans. The extent to which the transgenic disease animal model recapitulates the disease biology has been discussed extensively [220,221,222]. The amyloid deposits formed in the mouse models over the life-span of 1–2 years is structurally different from that in aged patients with AD [223]. In addition, conflicting observations regarding the degree of pathology across different studies have been reported, such as white matter impairment by DTI [92,224] and atrophy, probably due to the dynamic microstructural changes in various animal models [173].

The advantage of MRI molecular imaging stems from its superior resolution and improved signal-to-noise ratio, enabled by the development in high-field MRI and coil arrays. MRI provides versatile functional, structural, and molecular readouts and longitudinal, large field-of-view imaging capacity compared to other imaging modalities, such as two-photon microscopy, fluorescence molecular tomography, and optoacoustic microscopy [225,226,227,228,229,230]. The disadvantages and limitations of preclinical MRI methods include the following:

(1) Relatively low molecular sensitivity. Positron emission tomography (PET) provides excellent sensitivity in detecting receptors or molecules at the system level even in the pM–nM range, although the resolution is suboptimal for the small-animal brain [231,232,233]. In the case of in vivo MEMRI, the high dose of manganese chloride required might lead to increased risk of acute toxicity in the liver, heart, and kidney, and therefore this has not been widely applied. SPIO-nanoparticle-based contrast agents require much smaller amounts of injection compared to Gd-contrast agents due to the higher MR relaxivity.

(2) Requirements in terms of magnetic field and scanning time. For instance, Aβ plaque detection using endogenous CE-MRI methods requires a long scan time using high-field MRI to achieve sufficient image quality, which hinders the application for in vivo imaging.

(3) Confounders and limitations in functional imaging. fMRI in small animals is more challenging compared to that in the human brain [234,235]. For functional imaging, the imaging speed achievable by MRI is limited in reflecting the rapid neuronal processes compared to optical imaging [236]. In addition, the states of animal (whether they underwent imaging under awake or anesthetized free-breathing/ventilated condition) and the anesthetic used (e.g., isoflurane/ketamine) largely impact the functional readouts. Further application of fMRI in AD amyloidosis animals that are awake will improve the translational value of the results.

Additional knowledge gaps in MR imaging in AD amyloidosis animal models include the following:

(1) BBB integrity imaging: BBB impairment plays an important role in AD pathogenesis and neural dysfunction and is associated with cognitive decline [237,238,239,240,241,242]. However, conflicting data showing a lack of widespread BBB leakage have also been reported in several AD animal models [243]. Dynamic contrast-enhanced (DCE)-MRI has been used to detect the impaired BBB integrity in the hippocampus of patients with early AD [244]. Montagne et al. recently demonstrated impaired BBB integrity in 5 × FAD and *APOE4* mice compared to wild-type mice by using DCE-MRI assisted with Gd-DTPA [141] (Figure 2a–d) (Table 3). Dickie et al. reported that DCE-MRI failed to detect the difference between the TgF344 rats and wild-type rats at 18 months-of-age and that the increased BBB water permeability was detected by using multi-flip angle multi-echo (MFAME) water-exchange MRI in TgF344 rats compared to wild-type rats [245]. Using the same method, Dickie et al. further showed in a cross-sectional study that BBB water permeability was affected earlier in TgF344 rats (13–18 months-of-age) compared to that in wild-type rats in normal ageing (18–21 months-of-age) [246]. Further studies are required to establish non-invasive imaging tools for visualizing BBB integrity and to elucidate the degree of BBB impairment in AD animal models.

(2) Glymphatic system imaging: The glymphatic system has been shown to be important for the exchange of CSF with interstitial fluid and for the clearance of waste metabolites involving the aquaporin 4 water channel [247]. Emerging evidence suggests that glymphatic system dysfunction may contribute to the development of AD [247,248,249,250]. Recent studies by Da Mesquita et al. have shown impaired meningeal lymphatics in J20 and 5 × FAD mice compared to wild-type mice, which affected the microglia responses and the effect of anti-Aβ immunotherapy in these models [251,252]. DCE-MRI using Gd-based contrast agents have been developed to examine the brain-wide glymphatic system in both healthy and diseased brains in human [253,254] and in animal models [255,256,257,258,259]. In addition to DCE-MRI, DTI analysis along the perivascular space [260], phase alternate labeling with null recovery MRI [261], and Mn^2+^ nanoconstruct for MRI detection of CSF [262] are being developed for studying the interstitial and CSF flow kinetics in animal models. In vivo MR imaging for the glymphatic system in amyloidosis animal models remains to be demonstrated in future studies.

(3) Integrating MRI with plasma and CSF biomarkers: So far, few studies have evaluated the amyloidosis models by using MRI combined with peripheral biomarkers. Parent et al. demonstrated the link between functional connectivity abnormalities (rs-fMRI), hippocampal atrophy, and levels of CSF Aβ_1-42_ and cognitive deficits in McGill-R-Thy1-APP rats [116]. Using dynamic glucose-enhanced MRI, Huang et al. demonstrated an altered level of D-glucose in the brain parenchymal as well as in the CSF of aged APP/PS1 mice compared to wild-type mice [142]. The CSF and plasma biomarkers provide comprehensive readouts for the levels of Aβ, neuroinflammation, and neurodegeneration and may facilitate result extrapolation to human studies.

In summary, the multiplex MRI has significantly improved our understanding of the pathophysiology in AD amyloidosis animal models at a systematic level and has provided the possibility of non-invasive longitudinal monitoring of disease development.

## Figures and Tables

**Table 1:** MRI for detecting cerebral Aβ deposits in animal models of amyloidosis.

**MRI Using Endogenous Contrast**	**Animal**	**References**
T_2,_ relaxation time	5 × FAD, APP, APP/PS1, APPswe, PS mice	[29,30,59,60,61,62]
3D GRE, T_2_* 16.4 T	APP23 mice	[24]
T_2_*w GE, T_2_w SE	APP/PS1, APP_V717I_ mice	[23,39,63,64]
CESL	APP/PS1 mice	[27]
T_1_w, CE-MR	APP/PS1, PDAPP mice	[65]
3D GE T_2_*w	APP/PS1, PS1 mice	[66]
MTC	APP/PS1 mice	[25,26]
CRAZED, GE	APP_V717I_ × ADAM10-dn mice	[67]
QSM, SWI	Tg-SwDI, APP/PS1 mice	[32,33]
**MRI with Contrast Agents**	**Animal**	**References**
^19^F, BSA@FGQDs	AD mice	[49]
^19^F, TFMB	APP mice	[50]
^19^F, ^1^H, FSB	APPswe mice	[48]
^19^F, Shiga-Y51	APP/PS1 mice	[51]
^19^F, FMeC1 (Shiga-Y5)	APPswe mice	[52]
T_2_*w, sialic-acid-coated BSA MNP	APP/PS1 mice	[47]
T_2_*, Gd-DTPA-K6Aβ1-30	APP/PS1, APPswe mice	[36]
T_1_w, cyanine–Gd(III) complex	5 × FAD mice	[37]
T_2_*w GE Gd, Gd-DOTA, DOTAREM^®^,	APP_SL_/PS1_M146L_, APP/PS1_dE9_, APP23, APP_SwDI_, 3 × Tg, PS1 mice	[34,45]
T_2_*w GE, T_2_w SE, Gd-pF(ab’)24.1	APP/PS1 mice	[39]
T_2_*w, Gd-DTPA-Aβ1-40, MION	APP/PS1 mice	[35]
SWI MGE RARE, APP-SiMag	3 × Tg mice	[41]
T_2_*w, USPIO-PEG-Aβ1-42.B	APP/PS1 mice	[40]
T_1_w SE, ADx-001	APP/PS1 mice	[38]
T_2_*w, anti-AβPP SPIONs	APP/PS1 mice	[42]
T_2_*w, IgG4.1 NP	APPswe mice	[44]
T_2_*w GE, SPIO	APP23, APP23 × PS45 mice	[43]
T_1_w, HMON-Aβ40	APP/PS1 mice	[28]
T_2_*w MGE, MnCl_2_	5 × FAD mice, TgF344 rats	[46]
T_2_*w, Cur-MNPs	5 × FAD, APPswe mice	[57,58]
T_2_*w, W20/XD4-SPIONs	APP/PS1 mice	[56]
T_2_*w, NU4MNS Aβ oligomer	5 × FAD mice	[53]

BSA, bovine serum albumin; CE, contrast enhanced; CESL, chemical-exchange-sensitive spin-lock; CRAZED, COSY revamped with asymmetric z-GRE detection; Gd, gadolinium; GE, gradient echo; GRE, gradient recalled echo; MGE, multi-echo GRE; MION, monocrystalline iron oxide nanoparticles; MnCl2, Manganese(II) chloride; MNP, magnetic nanoparticle; MNS, magnetic nanostructures; MTC, magnetization transfer contrast imaging; NP, nanoparticle; PEG, polyethylene glycol; QSM, quantitative susceptibility mapping; RARE, rapid acquisition with relaxation enhancement; SE, spin echo; SWI, susceptibility-weighted imaging; USPION, ultrasmall superparamagnetic iron oxide nanoparticles; w, weighted.

**Table 2:** MRI for functional and neurochemical changes in animal models of amyloidosis.

Target	MRI	Animal	References
BOLD	rs-fMRI	APP^NL-F/NL-F^ ki mice	[90,96]
		APP/PS1 mice	[92,93,97,110,111]
		arcAβ mice	[88,92,112]
		TgCRND8 mice	[113]
		TgF344-AD rats	[99,114]
		PDAPP mice	[89]
		APPswe mice	[89,115]
		McGill-R-Thy1-APP rats	[116]
		3 × Tg mice	[91]
		E22ΔAβ mice	[92]
		TetO-APPswe/ind mice	[75]
CBF	ASL	Bigenic mice	[117]
		arcAβ mice	[104,105]
		3 × Tg mice	[118]
		APP DSL ki mice	[119]
		APP23 mice	[24,120]
		APP/PS1 mice	[101,102,111,120,121,122,123,124,125]
		J20 mice	[106]
		Tg-SwDI mice	[126,127]
		PS2APP mice	[108]
		5 × FAD mice	[128,129]
		TetOAPPswe, CAA mice	[130]
		APPswe mice	[107,131]
CBV	fMRI	BiAT mice	[117,132]
		APP23 mice	[43,133,134,135,136]
		arcAβ mice	[104,137]
		PDAPP mice	[138]
		APP/PS1 mice	[111,124]
		APPswe mice	[110]
		J20 mice	[139]
Synaptic funtion	MEMRI	3 × Tg mice	[85]
		APP/PS1-Ki mice	[78]
		J20 mice	[79]
		APPswe mice	[26,83,84,107]
		5 × FAD mice	[46,80]
		CVN-AD mice	[81]
		TgF344 rats	[46]
CMRO_2_	^17^OZTE	APPPS1 mice	[140]
BBB integrity	DCE	5 × FAD, APOE mice	[141]
Neurochemical profiles	DGE	APP/PS1 mice	[142]
	CEST	APP23 mice	[143]
		APP/PS1 mice	[144,145]
		5 × FAD mice	[129]
	^1^H MRS	TgF344 rats	[146]
		APP/PS1 mice	[121,147,148,149,150,151]
		5 × FAD mice	[152]
		3 × Tg mice	[153]
		APPswe mice	[115,154]
		TASTPM, APP/PS2/Tau mice	[155]

ASL, arterial spin labeling; BBB, blood–brain barrier; BOLD, blood-oxygen-level dependent; CBF, cerebral blood flow; CBV, cerebral blood volume; CE, contrast enhanced; CEST, chemical exchange saturation transfer; CMRO_2_, cerebral metabolic rate of oxygen consumption; DCE, dynamic contrast enhanced; DGE, dynamic glucose enhanced; fMRI, functional magnetic resonance imaging; MEMRI, manganese-enhanced magnetic resonance imaging; MRS, magnetic resonance spectroscopy; ZTE, zero echo time.

**Table 3:** MRI for detecting atrophy, white matter integrity, and cerebral vasculature alterations in animal models of amyloidosis.

	MRI	Animal	References
Atrophy	T_2_	APP/J20 mice	[170]
		APP/PS2/Tau mice	[155]
		TASTPM mice	[155,171]
		APP/PS1 mice	[102,122,149,172,173]
		McGill-R-Thy1-APP rats	[174]
		PDAPP mice	[175,176]
		APP-Au mice	[177]
		3 × Tg mice	[91,178,179]
		APPswe mice	[131]
		APP/PS1KI mice	[180]
		APP/TTA mice	[181]
White matter integrity	DKI	APP/PS1 mice	[182]
		3 × Tg mice	[183]
	qMTI	APPswe mice	[184]
	DTI	TgF344 rats	[185]
		APPswe mice	[184,186,187,188]
		PDAPP mice	[189]
		App^NL-G-F^ knock-in mice	[71]
		APP/PS1 mice	[121,190,191,192,193,194]
		APP23 mice	[195]
		3 × Tg mice	[91,183,196,197,198,199]
		TgCRND8 mice	[113]
		APP/TTA mice	[81,181]
		CVN-AD mice	[95]
		5 × FAD mice	[129]
Microbleeds, iron	SWI, QSM	arcAβ mice	[112,167,200]
		APP/PS1 mice	[168]
		CVN-AD mice	[81]
	T_2_*	Tg SwDI mice	[166]
	T_2_*w	APP23 mice	[165]
Inflammation	T_2_*w, MPIOs-αVCAM-1	APP/PS1 mice	[201]
Cerebrovasculature	QUTE-CE	APOE4 rats	[202]
	DWI	5 × FAD mice	[203]
	MRA	arcAβ mice	[156,167,169]
		APP/PS1 mice	[109,204]
		APP23 mice	[134,136]
		APPswe mice	[205]
	MION	5 × FAD mice	[203]
	MRE	5 × FAD mice	[206]
		APP/PS1 mice	[207]
		APP23 mice	[208]

CE, contrast enhanced; CV, cerebral viscoelastic; DKI, diffusional kurtosis imaging; DTI, diffusion tensor imaging; DWI, diffusion-weighted imaging; MFAME, multi-flip angle multi-echo; MION, monocrystalline iron oxide nanoparticle; MPIOs, micro-sized particles of iron oxide; VCAM-1, vascular cell adhesion molecule-1; MRA, magnetic resonance angiography; MRE, magnetic resonance elastography; qMTI, quantitative magnetization transfer imaging; QSM, quantitative susceptibility mapping; QUTE-CE, quantitative ultrashort time-to-echo, contrast enhanced; SWI, susceptibility-weighted imaging; w, weighted.
